# Association of Psoriasis With Anxiety and Depression: A Case–Control Study in Chinese Patients

**DOI:** 10.3389/fmed.2021.771645

**Published:** 2021-12-24

**Authors:** Danrong Jing, Hui Xiao, Minxue Shen, Xiang Chen, Xi Han, Yehong Kuang, Wu Zhu, Yi Xiao

**Affiliations:** ^1^Department of Dermatology, Xiangya Hospital, Central South University, Changsha, China; ^2^Hunan Key Laboratory of Skin Cancer and Psoriasis (Xiangya Hospital), Central South University, Changsha, China; ^3^Hunan Key Laboratory of Skin Cancer and Psoriasis, Central South University, Changsha, China; ^4^Department of Social Medicine and Health Management, Xiangya School of Public Health, Central South University, Changsha, China; ^5^ULink College Guangzhou, Guangzhou, China

**Keywords:** psoriasis vulgaris, anxiety, depression, comorbidities, case control study

## Abstract

**Background:** Patients with psoriasis are prone to suffer from anxiety and depression during their lifetime. This study aimed to investigate the association of psoriasis with anxiety and depression in Chinese patients.

**Methods:** A case-control study in Chinese patients with psoriasis vulgaris and healthy controls was conducted. Clinical information based on patient-reported, clinical information, and reliable structured questionnaires were collected. Multivariable logistic regression was used to investigate the associations, in terms of adjusted odds ratios (AORs).

**Results:** We continuously selected 1,571 patients who were firstly diagnosed with psoriasis vulgaris, and 1,571 healthy controls, matched by age and sex. The risk of depression in the psoriasis vulgaris group was higher than that in the healthy controls (AOR = 1.30, *P* = 0.047), while no differences were found in the risk of anxiety between the two groups (AOR = 1.18, *P* = 0.381). Subgroup analysis by disease onsets showed that late-onset psoriasis (LOP) was significantly associated with a higher risk of anxiety (AOR = 1.47, *P* = 0.033) and depression symptoms (AOR = 1.85, *P* = 0.012) but not with early-onset psoriasis (EOP). Subgroup analysis by disease severity indicated that no difference was observed in the associations of mild psoriasis vulgaris, moderate-to-severe psoriasis vulgaris with anxiety and depression.

**Conclusion:** Patients with psoriasis vulgaris were more likely to develop depression compared with the general population. LOP patients were positively associated with anxiety and depression. We believe the screening of emotional disorders should be included in the daily management of psoriasis patients.

## Introduction

Psoriasis is a chronic inflammatory skin disease associated with several medical comorbidities such as psychosocial disorders, psoriatic arthritis, and cardiometabolic syndrome (1). The prevalence rate is known between 0.1 and 11.4% globally, and approximately 0.47% in China (1–3). Psoriasis can present at any age, but it mainly affects the populations before the age of 35 years (4). Psoriatic lesions usually occurred in the visible skin areas involved the scalp, limbs, and extensor surfaces such as the elbows and knees (5). The appearance of the affected skin is believed to impact the patient's psychosocial wellbeing, as low self-esteem, embarrassment, sexual dysfunction, and even suicidal ideation (6). Previous reports found early-onset psoriasis (EOP), meaning first presented psoriatic lesions at and prior to the age of 40 years, comprises approximately 70% of all psoriasis. And late-onset psoriasis (LOP), which presents after 40 years old (7, 8). Patients with EOP were more likely to have a family history, more extensive body surface involvement, and strongly associated with HLA-C^*^06, while patients with LOP tend to be sporadic, accompanied with numerous complications, such as diabetes, high blood pressure, dyslipidemia, and obesity (9).

Previous studies found that patients with psoriasis commonly occured anxiety and/or depression, and moderate-to-severe psoriasis were more likely to suffer from physical or mental impairments (10). In turn, this emotional condition also can exacerbate psoriasis and even impair the treatment outcomes (11–13). Recently, some studies demonstrated that anxiety and depression also belonged to chronic inflammatory disease (14, 15). Thus, early identification of the emotional disorder is important for improving the health-related quality of life and clinical outcomes. However, to our knowledge, relevant data from China about the association of psoriasis with anxiety and depression are rare. Moreover, little evidence has been shown that whether the association between psoriasis and depression/anxiety can be different among LOP patients and EOP patients.

Therefore, in this case-control study, we enrolled psoriasis vulgaris patients and healthy control participants, and assessed the relationship of psoriasis with the symptoms of anxiety and depression in Chinese patients.

## Methods

### Study Design and Participants

This case-control study was carried out in Chinese patients with psoriasis vulgaris and healthy controls in 2018. Each recruited patient who diagnosed with psoriasis vulgaris was confirmed by two or more professors, and had not received any treatment. The patients were divided into two groups according to their disease severity based on the Psoriasis Area and Severity Index (PASI): PASI score <10 can be classified as mild psoriasis, PASI score≥10 is defined as moderate-to-severe psoriasis (16, 17). The healthy controls were from the Hunan Civil Servant Cohort (18). Additionally, we categorized patients who developed psoriatic lesions at or younger than 40 years old as early-onset, and older than 40 years old as the late-onset (7). The healthy controls who had been reported a history of psoriasis were excluded. The ratio of psoriasis vulgaris and healthy controls were 1:1, matching for sex and age (±1 years). The study followed the guidelines established in the Declaration of Helsinki and was approved by the Medical Ethical Committee of Xiangya Hospital, Central south university (Approval. No. 201709993). All participants have signed the informed consent prior to enrollment.

### Data Collection

We collected the demographic information (such as age, sex, education level, cigarette smoking, and alcohol drinking) from all participants by face-to-face interview. The psoriasis area severity index (PASI) score was applied to examine the dermatological status of included patients: mild (PASI <10), moderate-to-severe (PASI ≥10). To assess the symptoms of generalized anxiety, we used the Generalized Anxiety Disorder 7-Item (GAD-7) scale, GAD-7 was the most widely used self-assessment tool for evaluating anxiety (19, 20), the scale of GAD-7 has acceptable diagnostic accuracy when the cut-off scores was ≥ 8 points (sensitivity = 0.97, specificity = 0.75) (21). And the 9-item Patient Health Questionnaire (PHQ-9) scale was used to evaluating the depressive symptoms, PHQ-9 was proved to be a reliable self-report questionnaire for evaluating depression (22), the sensitivity and specificity of PHQ-9 (cut-off scores was ≥ 8 points) was 85.7 and 80.2% respectively (23). The cutoff point for both scales was ≥8 points. The Chinese versions of the tools in our sample were validated previously (24, 25).

### Statistical Analysis

Continuous variables with normal distribution were presented as mean ± standard deviation (SD) and compared with analysis of variance (ANOVA). Categorical variables were summarized as counts (percentages) and compared using the chi-square test or Fisher's exact test. The associations of psoriasis vulgaris with outcomes were assessed by multivariable logistic regression with adjustments for potential confounders. Odds ratio (OR) and 95% confidence interval were used to present the effect size of the associations. *P*-value < 0.05 was considered to be statistically significant. The data were analyzed with SPSS 25 (IBM, SPSS Statistics 25).

## Results

### Characteristics of the Cases and Healthy Controls

There were 1,571 patients with psoriasis vulgaris and 1,571 healthy control subjects in our study. Among the psoriasis vulgaris group, there were 1,002 cases of early-onset and 569 cases of late-onset. The mean age of the patients with psoriasis vulgaris was 44.3 ± 12.8 years, and 951 (60.5%) were male. No significant differences were observed in terms of age or gender between the two groups (*P* = 0.001). The proportion of education level, annual income and smoking status was significantly higher among healthy control subjects compared to those with psoriasis patients (*P* < 0.001). In addition, psoriasis patients also had significantly higher proportion of alcohol drinking behaviors than healthy control subjects (*P* < 0.05). The demographic characteristics of the patients with psoriasis vulgaris and the healthy controls are presented in Table 1.

**Table 1:** Characteristics of the cases and healthy controls.

	**Psoriasis vulgaris** **(*n* = 1,571)**	**Control** **(*n* = 1,571)**	***P***
	***n* (%)**	***n* (%)**	
Age (mean ± SD)	44.3 ± 12.8	44.3 ± 12.8	1.000
Male	951 (60.5)	951 (60.5)	1.000
Education			
Primary/middle school	726 (46.2)	32 (2.7)	<0.001
High school	362 (23.0)	127 (8.1)	
College or above	483 (30.7)	1,402 (89.2)	
Income (¥)^a^			
<50,000	562 (35.8)	225 (14.3)	<0.001
50,000–109,999	933 (59.4)	475 (30.2)	
110,000–199,999	76 (4.8)	522 (33.2)	
>200,000	0 (0.0)	349 (22.2)	
Smoking			
Current	537 (34.2)	238 (15.2)	<0.001
No/Former	1,034 (65.8)	1,333 (84.8)	
Alcohol drinking			
NO	1,251 (76.2)	1,197 (79.6)	0.031
Yes	320 (23.8)	374 (20.4)	

a *1 ¥ = US $0.15*.

### Association of Psoriasis Vulgaris With Anxiety and Depression

The result of the crude ORs and adjusted ORs (AORs) of the association of psoriasis vulgaris with anxiety and depression were shown in Table 2. In the psoriasis vulgaris group, 326/1571 (20.75%) cases completed the GAD-7 and PHQ-9. 1505/1571 (95.80%) cases finished two questionnaires in the control group. The risk of depression in the psoriasis vulgaris group was 1.30 times (AOR = 1.30; 95% CI 1.01–1.79; *P* = 0.047) higher than that in the healthy controls. However, significant differences were not found in the risk of anxiety between two groups (AOR = 1.18; 95% CI 0.93–1.75; *P* = 0.381). This result concluded that statistically significant associations between psoriasis vulgaris and depression.

**Table 2:** The association between depression and anxiety of the participants among two groups based on the questionnaires.

**Item**	**Control**	**Psoriasis vulgaris**			
	***n* (%)**	***n* (%)**	**OR (95% CI)**	**AOR (95% CI)^a^**	***P***
Anxiety (GAD-7^b^ ≥8)	372 (21.1)	94 (28.8)	1.23 (0.95–1.61)	1.18 (0.93–1.75)	0.381
Depression (PHQ-9^c^ ≥8)	451 (30.0)	118 (36.2)	1.32 (1.03–1.70)	1.30 (1.01–1.79)	0.047

a *Adjusted for age, sex, annual income, educational level, smoking, alcohol, and drinking*.

b *GAD-7, Generalized Anxiety Disorder-7;*

c *PHQ-9, Patient Health Questionnaire-9; P value for adjusted OR, estimated from the multivariable logistic regression model*.

*OR, unadjusted odds ratio; AOR, adjusted odds ratio; CI, confidence interval*.

### Subgroup Analysis by Disease Onset

The analysis of the subgroup analysis for the association of psoriasis vulgaris with anxiety and depression in early- and late-onset psoriatic lesions groups were reported in Table 3. The risk of late-onset psoriasis vulgaris with anxiety was 1.47 times than that in the control group (95%CI 1.05–2.34; *P* = 0.033), while this phenomenon was not observed in the early-onset psoriasis vulgaris (AOR = 1.06; 95%CI 0.74–1.52; *P* = 0.742). Late-onset psoriasis vulgaris was significantly associated with a higher risk of depression symptoms as compared to the control group (AOR = 1.85; 95% CI 1.15–3.02; *P* = 0.012), while early-onset psoriasis vulgaris was not associated significantly with the depression symptoms (AOR = 1.08; 95%CI 0.77–1.50; *P* = 0.656). The unobvious links between early-onset psoriasis vulgaris and anxiety, depression may be explained by the longer course of patients with early-onset psoriasis.

**Table 3:** Subgroup analysis for the association between depression and anxiety the participants among two groups by Disease Onset.

**Item**	**EOP**					**LOP**				
	**Case** **(*n* = 228)**	**Control** **(*n* = 996)**	**OR (95%CI)**	**AOR (95%CI)^a^**	**P**	**Case** **(*n* = 98)**	**Control** **(*n* = 563)**	**OR (95%CI)**	**AOR (95%CI)^a^**	**P**
	***N* (%)**	***N* (%)**				***N* (%)**	***N* (%)**			
Anxiety (GAD-7^b^ ≥8)	68 (29.8)	284 (29.3)	1.03 (0.75–1.41)	1.06 (0.74–1.52)	0.742	26 (26.5)	88 (16.4)	1.84 (1.11–3.04)	1.47 (1.05–2.34)	0.033
Depression (PHQ-9^c^ ≥8)	83 (36.4)	337 (34.8)	1.07 (0.80–1.45)	1.08 (0.77–1.50)	0.656	35 (35.7)	114 (21.3)	2.06 (1.30–3.27)	1.85 (1.15–3.02)	0.012

a *Adjusted for age, sex, annual income, educational level, smoking, alcohol, and drinking;*

b *GAD-7, Generalized Anxiety Disorder-7;*

c *PHQ-9, Patient Health Questionnaire-9;*

*P value for adjusted OR, estimated from the multivariable logistic regression model*.

*OR, unadjusted odds ratio; AOR, adjusted odds ratio; CI, confidence interval*.

### Subgroup Analysis by PASI

The patients with psoriasis vulgaris were divided into mild and moderate-to-severe groups, and the ratio of mild psoriasis vulgaris and moderate-to-severe psoriasis vulgaris were 1:1, matching for the same composition of sex and age. After the matching, each of two groups involved 424 patients. The demographic characteristics of the two groups were shown in Table 4. Only the education level of moderate-to-severe group was significantly lower than that the mild group (*P* < 0.001), and no statistically significant difference was found in other demographic indicators.

**Table 4:** Subgroup analysis for the association between depression and anxiety the participants among two groups by PASI score.

	**Mild psoriasis vulgaris (*n* = 424)**	**Moderate-to-severe psoriasis vulgaris (*n* = 424)**	***P***
	***n* (%)**	***n* (%)**	
Age (mean ± SD)	40.6 ± 14.5	40.6 ± 14.5	1.000
Male	297 (70.0)	297 (70.0)	1.000
PASI	4.6 ± 2.8	18.1 ± 8.1	<0.001
Education			
Primary/middle school	186 (43.9)	220 (51.9)	0.003
High school	92 (21.7)	105 (24.8)	
College or above	146 (34.4)	99 (23.3)	
Income (¥)^a^			
<50,000	157 (37.0)	183 (43.2)	0.057
50,000–100,000	243 (57.3)	228 (53.8)	
>100,000	24 (5.7)	13 (3.1)	
Marital status			
Unmarried	68 (16.0)	63 (14.9)	0.750
Married	354 (83.5)	360 (84.9)	
Divorced/ Widowed	2 (0.5)	1 (0.2)	
Occupation			
Worker	42 (9.9)	48 (11.3)	0.288
Farmer	48 (11.3)	65 (15.3)	
Technicians	59 (13.9)	52 (12.3)	
Sales workers	20 (4.7)	25 (5.9)	
Student	48 (11.3)	39 (9.2)	
Retired	65 (15.3)	72 (17.0)	
Self-employed	38 (9.0)	24 (5.7)	
Other	104 (24.5)	99 (23.3)	

a *1 ¥ = US $0.15*.

Table 5 shows the subgroup analysis of the associations of psoriasis vulgaris with anxiety and depression in mild or moderate-to-severe psoriasis vulgaris. 53/193 (27.5%) mild psoriasis cases and 67/193 (30.4%) moderate-to-severe cases were reported anxiety, 67/92 (34.7%) mild psoriasis cases and 34/92 (37.0%) moderate-to-severe cases were reported depression. When we compared the risk of anxiety and depression between two groups, no difference was observed in the associations of mild psoriasis vulgaris, moderate-to-severe psoriasis vulgaris with anxiety (AOR = 1.15; 95% CI 0.67–1.99; *P* = 0.612), depression (AOR = 1.08; 95% CI 0.64–1.81; *P* = 0.781).

**Table 5:** Subgroup analysis for the association between depression and anxiety the participants among two groups by PASI score.

**Item**	**Mild (PASI <10) (*n* = 193)**	**Moderate-to-severe (PASI≥10) (*****n*** **=** **92)**			
	***n* (%)**	***n* (%)**	**OR (95% CI)**	**AOR (95% CI)^a^**	***P***
Anxiety (GAD-7^b^ ≥8)	53 (27.5)	28 (30.4)	1.16 (0.67–1.99)	1.15 (0.67–1.99)	0.612
Depression (PHQ-9^c^ ≥8)	67 (34.7)	34 (37.0)	1.10 (0.66–1.85)	1.08 (0.64–1.81)	0.781

a *Adjusted for age, sex, annual income, educational level, smoking, alcohol, and drinking;*

b *GAD-7, Generalized Anxiety Disorder-7;*

c *PHQ-9, Patient Health Questionnaire-9;*

*P value for adjusted OR, estimated from the multivariable logistic regression model*.

*OR, unadjusted odds ratio; AOR, adjusted odds ratio; CI, confidence interval; PASI, the psoriasis area severity index*.

## Discussion

Based on reliable structured questionnaires, we compared the risk of anxiety and depression in psoriasis vulgaris in Chinese patients. The finding of this study indicated that psoriasis vulgaris have a higher risk of depression compared with the healthy controls, while the difference was not statistically significant in anxiety. With regards to the onset age and severity of the disease, we detected the symptoms of anxiety and depression were more common in Late-onset Psoriasis but not Early-onset psoriasis. And we found an unobvious impact of psoriasis severity on anxiety and depression.

Numerous previous studies proved that psoriasis was associated with depression. A prospective cohort study in US pointed out that patients with psoriasis were more prone to develop depression than those without psoriasis (26). Another population-based cohort study indicated psoriasis patients were at a higher risk of developing depression compared to the general population (27). Our analysis supports patients with psoriasis have a statistically significant risk factor for developing depression. However, we found that there was not a statistically significant risk of anxiety in psoriasis patients when compared with the healthy control, which was different from other studies (28, 29). The difference in the risk of anxiety may be explained by the sample size.

Previous studies suggested EOP patients were more likely to be anxious and depressive (30, 31). However, we found LOP was significant associated with anxiety and depression, but no significant correlation was observed in EOP. The explanation of our results may be that LOP patients were more commonly reported the symptoms of itching, sensitive skin, and burning and worry about the disease, which can result in psychological burdens (32). Moreover, LOP patients were common with chronic inflammatory comorbidities, such as type 2 diabetes, obesity, autoimmune thyroiditis, which can reduce the quality of life and increase the cost of treatment (33, 34). In addition, the status of depression has been believed to plays roles in the onset or exacerbation of psoriasis (35). Anxiety-depression is now considered a chronic inflammatory disease, and LOP is thought to more related to prolonged inflammation. Abundant studies have indicated that pro-inflammatory cytokines can be released in both depression and psoriasis (36). It has found that patients with psoriasis showed elevated TNF-a, IL-12, IL-23, IL-17 (37), and high level of TNA-a, IL-2, IL-6, IL-17, IL-23 was found in depression (38, 39). In addition, patients with other inflammatory diseases such as type 2 diabetes, obesity, autoimmune thyroiditis also associated with depression (40–42). Psoriasis also showed a positive association with the above inflammatory diseases, reminding that chronic inflammation status may play a role between psoriasis and anxiety-depression (43). Psoriasis patients with severe depressive symptoms were found higher levels of inflammatory cytokines in the peripheral blood such as TNF-α, IL-6, and IL-17 than those with low depressive patients (44–47). Recently, the gut-brain-skin axis was thought to associated with the overlapping mechanisms between psoriasis and depression. Psoriasis and depression can lead to the dysregulation of the gut microbiota, induce gut permeability and bacterial migration, then increases pro-inflammatory cytokines such as IL-β, IL-6, TNF-α and IFN-γ (35). These cytokines were contribute to the differentiation of Th17 cells and induce further inflammation in psoriasis and depression (48, 49). And it also can lead to the activation of the HPA axis and increase the release of glucocorticoid, then promote the inflammatory response in psoriasis and depression (35). Thus, the status of depression-anxiety has been believed to plays roles in the onset or exacerbation of psoriasis (35). Taken together, the above explanations may be supported that LOP is more related to the mental disorder.

The correlation between the severity of psoriasis and anxiety, depression was controversial. Kurd et al. showed that the relative risks of depressive symptoms in patients with severe psoriasis were higher than mild psoriasis (50). While a hospital-based case-control study by Golpour et al. revealed that no association was found between clinical severity and symptoms of psoriasis and anxiety and/or depression (51). And Fortune DG et al. has reported that the magnitude of the association between the clinical severity of patients with psoriasis and depression tends to be slight (52). This study support no significant correlation between the severity of psoriasis and anxiety, depression (53).

There are some limitations in this study. Firstly, the main drawback is the design of our study, which makes it difficult to determine the causality because the chronological sequence of exposure and disease outcome was unclear. Secondly, this study is conducted in a group of participants in a single-center, which may limit the broader generalization of the results. And our study also has some strengths. First of all, the exact matching with age and gender reduces the bias caused by demographic factors. Afterwards, the sample size of this study is relatively large when compared with other epidemiological studies.

## Conclusion

Psoriasis vulgaris in Chinese patients were more likely to developed depression. Late-onset patients with psoriasis were more likely to combined with anxiety and depression. Thus, clinicians should pay attention to the emotion in psoriasis patients, and routine examinations should include the screen of psychological disorders in the daily management.

## Data Availability Statement

The original contributions presented in the study are included in the article/supplementary material, further inquiries can be directed to the corresponding authors.

## Ethics Statement

The studies involving human participants were reviewed and approved by the Medical Ethical Committee of Xiangya Hospital, Central South University (Approval No. 201709993). The patients/participants provided their written informed consent to participate in this study.

## Author Contributions

YX, HX, and XH: data collection and writing. MS and YK: study design and funding. WZ and DJ: data analysis. XC and WZ: review the manuscript. All authors: data interpretation, revision, and final approval.

## Funding

This work was supported by the National Natural Science Foundation of China (81974479, 81773329, 82103737, and 82173426), the National Key Research and Development Project of China Precision Medicine Initiative (2016YFC0900802), and the Program of Introducing Talents of Discipline to Universities (111 Project, No. B20017). The funders did not participate in this study.

## Conflict of Interest

The authors declare that the research was conducted in the absence of any commercial or financial relationships that could be construed as a potential conflict of interest.

## Publisher's Note

All claims expressed in this article are solely those of the authors and do not necessarily represent those of their affiliated organizations, or those of the publisher, the editors and the reviewers. Any product that may be evaluated in this article, or claim that may be made by its manufacturer, is not guaranteed or endorsed by the publisher.
